# Association of postoperative atrial fibrillation with higher dosing ratios of protamine-to-heparin

**DOI:** 10.1051/ject/2023003

**Published:** 2023-03-24

**Authors:** Yasuharu Yamada, Junzo Iemura, Atushi Kambara, Noboru Tateishi, Yuji Kozaki, Masako Yamada, Junko Maruyama, Eiichi Azuma

**Affiliations:** 1 Department of Clinical Engineering, Faculty of Medical Engineering, Suzuka University of Medical Science Mie 513-8670 Japan; 2 Department of Cardiovascular Surgery, Okanami General Hospital Mie 518-0121 Japan; 3 Division of Clinical Engineering, Okanami General Hospital Mie 518-0121 Japan; 4 Department of Clinical Engineering, Mie University Hospital Mie 514-0001 Japan

**Keywords:** Postoperative atrial fibrillation (POAF), Calcium, Protamine-to-heparin ratio, Cardiopulmonary bypass, Anticoagulation

## Abstract

*Background*: Postoperative atrial fibrillation (POAF) is defined as new-onset AF in the immediate postoperative period. The relatively high incidence of POAF after cardiac surgery is well described, but pathophysiological mechanisms underlying the initiation, maintenance, and progression of POAF may be multifactorial and have not yet been comprehensively characterized. One of the mechanisms includes altered Ca^2+^ kinetics. Accumulating evidence has suggested that altered atrial cytosolic calcium handling contributes to the development of POAF, protamine reversibly modulates the calcium release channel/ryanodine receptor 2 (RyR2) and voltage-dependent cardiac RyR2. However, it is currently unknown whether such abnormalities contribute to the arrhythmogenic substrate predisposing patients to the development of POAF. *Methods*: We have retrospectively analyzed 147 patients who underwent cardiac surgery with cardiopulmonary bypass support. Of these, 40 patients were excluded from the analysis because of pre-existing AF. All patients received heparin followed by protamine at different dosing ratios of protamine-to-heparin, depending on the periods studied. *Results*: The dosing ratio of protamine-to-heparin = 1.0 was compared with higher dosing ratios of protamine-to-heparin >1.0 up to 1.7. POAF developed in 15 patients (15/107 = 14%), of these, 5 out of 57 patients (33.3%) in the dosing ratio of protamine-to-heparin = 1.0 and 10 out of 35 patients (66.7%) in the higher dosing ratios of protamine-to-heparin. Statistical significance was observed in patients with higher dosing ratios of protamine-to-heparin, compared with the dosing ratio of protamine-to-heparin = 1.0 (odds ratio = 3.890, 95% CI = 1.130–13.300, *p*-value = 0.031). When types of diseases were analyzed in terms of higher dosing ratios of protamine-to-heparin, only valvular disorders were significantly associated with POAF (*p* = 0.04). *Conclusions*: Protamine is clinically utilized to reverse heparin overdose and has been shown to display immunological and inflammatory alterations. However, its association with POAF has not been reported. Our results provide evidence that higher dosing ratios of protamine-to-heparin may increase the incidence of POAF.

## Introduction

Postoperative atrial fibrillation (POAF) complicates 20–40% of cardiac surgical procedures [1]. Typical features include onset at 2–4 days postoperatively, episodes that are often fleeting, and a self-limited time course. Associated adverse consequences of POAF include hemodynamic instability, increased risk of stroke, lengthened hospital and intensive care unit stays, and greater costs [1].

The mechanisms of POAF are complex and incompletely defined, but include intraoperative and postoperative phenomena, such as inflammation, sympathetic activation, hypoxia, acidosis, oxidative stress, electrolyte abnormalities, and intra-atrial conduction delay that combine to trigger atrial fibrillation, often in the presence of pre-existing factors, making the atria vulnerable to atrial fibrillation induction and maintenance [1, 2]. To prevent POAF, preoperative beta-blocker (propranolol, carvedilol plus N-acetyl cysteine) use is associated with a reduced incidence of postoperative AF, but not major adverse events such as death, stroke, or acute kidney injury [3]. Data for other interventions such as statins, magnesium, colchicine, posterior pericardiotomy, atrial pacing, and corticosteroids are not robust. Two large randomized controlled studies showed no significant effect of i.v. steroids on the incidence of postoperative AF after cardiac surgery [4]. Thus, other modalities may be needed, although the molecular events initiating AF remain uncertain.

Considering that cytosolic Ca^2+^ handling is a major determinant of cardiac contractile function, Fakuade et al. hypothesized that alterations in cellular Ca^2+^ dynamics contribute to impaired atrial contractility in patients who proceed to develop POAF [5]. They found that reduced sarcoplasmic reticulum (SR) Ca^2+^-ATPase (SERCA)-mediated Ca^2+^ reuptake into SR is a major contributor to impaired preoperative atrial contractile function [5]. Ryanodine receptors (calcium-induced calcium release channels; RyR) play a crucial role in most cell types, including muscle cells, neurons, and epithelial cells. They mediate the release of calcium ions from the endoplasmic/sarcoplasmic reticulum into the cytosol and thereby convert a number of extracellular stimuli into intracellular calcium signals [6]. RyRs are large tetrameric proteins that show sequence similarity with inositol 1,4,5-trisphosphate (IP3)-gated calcium channels of the endoplasmic/sarcoplasmic reticulum, but they are distinct in their biophysical and pharmacological properties. For example, highly negatively charged polyanions such as heparin increased the activity of RyRs, whereas it decreased the activity of IP3 receptors [7]. Diaz-Sylvester and Copello found that a lower dose of protamine activated cardiac RyR2 [8]. Shan et al. in mouse models with known RyR2 mutations and normal cardiac structure and function, the diastolic SR Ca^2+^ leak via RyR2 lead to Ca^2+^ waves and possible re-entry loops that trigger atrial tachycardia and AF [9].

In STS/SCA/AmSECT (Society of Thoracic Surgeons, The Society of Cardiovascular Anesthesiologists, and the American Society of ExtraCorporeal Technology) clinical practice guidelines published in 2018 [10], recommendations were written in the reversal of anticoagulation during cardiac operations. Methods of heparin reversal are multiple and controversy exists regarding the optimal strategy. Traditional methods administer heparin based on body weight and protamine based on the amount of heparin administered. An important part of the operation is to adequately remove all of the heparin effects at the end of the operation. There are at least three methods commonly used to detect residual heparin effect after protamine reversal. Among them, the ACT-based strategy is commonly used, where activated clotting time (ACT) measurement is kept greater than 480 s which seemed to be appropriate during cardiac surgical procedures in this study. Class IIa recommendation in the guidelines included limiting the ratio of protamine/heparin to less than 2.6 mg protamine/100 Units of heparin, since total doses above this ratio inhibit platelet function, prolong ACT, and increase the risk of bleeding [10].

Here, we have retrospectively reviewed 147 patients who underwent cardiac surgery with cardiopulmonary bypass. All these patients received heparin followed by protamine at different dosing ratios of protamine-to-heparin, depending on the periods used. We have found that higher dosing ratios of protamine-to-heparin increased the incidence of POAF.

## Materials and methods

Data Collection was conducted as a retrospective cohort study to clarify the prevalence and incidence of POAF in the patients who received cardiac surgery. The study protocol conformed to the Declaration of Helsinki and was approved by the institution’s ethics committee in Okanami General Hospital, Iga-City, Mie Prefecture, Japan. Okanami General Hospital is a secondary medical center in the Iga district. The study database was from the Okanami Hospital Medical Database which was composed of detailed medical and procedural information.

Atrial fibrillation and its occurrence time were identified by the diagnosis of health records and electrocardiograms. Comorbidities including hypertension, diabetes mellitus, hyperlipidemia, heart failure, coronary artery disease, myocardial infarction, chronic obstructive pulmonary disease, peripheral arterial occlusive disease, and chronic kidney disease were also coded from the records. Prescription information was categorized into antiarrhythmic agents, beta-blockers, angiotensin-converting enzyme inhibitors (ACEI), angiotensin receptor blockers (ARBs), mineralocorticoid-receptor antagonists, calcium blockers, digitalis preparation, sodium channel blocker, and anticoagulants. The open heart procedures included coronary artery bypass grafting, valvuloplasty, and valve replacement. The echocardiographic data and outcomes including transient ischemic attack, ischemic stroke, hospitalization, and mortality were also obtained from the electronic health records. Activated clotting time (ACT) was measured using Hemochron^®^ (Accriva Diagnostics, San Diego, CA, USA) from April 1, 2002, to March 12, 2015, and ACT Plus^®^ (Medtronic, Minneapolis, MN, USA) from March 13, 2015, and thereafter. ACT was determined before CPB, and then every 30 min. Additional heparin (1000–2000 IU) was administered if necessary. An ACT target of ≥ 480 s (≥ 480 s up to 600 s) was set in the hospital. All patients received unfractionated heparin (1000 international units/mL) and protamine (10 mg/mL), both are purchased from Mochida Pharmaceutical Co. Ltd. (Tokyo, Japan). All patients received initial heparin doses of 300 IU/kg. The dosing ratios of protamine-to-heparin were determined by the volume-based method in the clinical setting, that is, the dosing ratio of protamine-to-heparin = 1.0 represents 1 mL protamine to 1 mL heparin of the first heparin bolus. This ratio is equal to 1 mg of protamine to 100 international units of heparin. The dosing ratios of protamine-to-heparin >1.0 (up to 1.7) were used from February 2004 to September 2009 and the dosing ratio of protamine-to-heparin = 1.0 from October 2009 and thereafter. The change of dosing ratios of protamine-to-heparin was made according to the instructions of the pharmaceutical company.

*Patient Selection*. In this study, all patients above 18 years of age undergoing first-time open-heart surgery at Okanami General Hospital from February 1, 2004, to December 13, 2021, were included for analysis (*n* = 147). In this retrospective study, we did not include off-pump CABG cases (*n* = 91) who did not receive protamine. Thus, the annual surgical volume is 14–16 patients per year in the Department of Cardiovascular Surgery, Okanami General Hospital. Patients with previous documented AF (*n* = 40), who received anticoagulant therapy within 6 months before the open heart surgery or during the follow-up period, could not survive the open-heart procedures, and could not receive regular follow-up at the out-patient clinics were all excluded from the analysis. The definition of POAF was new-onset AF, which is sustained for over 30 s, and detected by either continuous telemetry in the intensive care unit, standard 12-lead electrocardiogram, or implanted devices. All medical records were followed until their last clinical visit, repeat cardiac surgery or death. The index date of outcomes was defined as the date of diagnosis.

*Statistical analysis*. All statistical analyses were performed using EZR software (available free of charge at the website of Saitama Medical Center, Jichi Medical University, Saitama, Japan) [11], which is a graphical user interface for the program R (The R Foundation for Statistical Computing, Vienna, Austria). The software is a modified version of R commander software (version 2.4-0) that contains additional statistical functions frequently used in biostatistics. Results were expressed as odds ratios (ORs) with 95% confidence intervals (CIs) from logistic regressions. Fischer’s exact test and chi-square test were used to analyze frequency distributions. The t-test was used to determine whether differences in the means of two sets of samples were significant. A *P-*value less than 0.05 was considered significant.

## Results

A total of 147 patients received cardiac surgery between February 1, 2004, to December 13, 2021, of which 40 patients were excluded because of pre-existing AF. After excluding patients with pre-existing AF, a total of 107 patients were enrolled in this cohort. Table 1 reports odds ratios (ORs) and 95% confidence intervals from logistic regression analysis of POAF incidence. As shown in Table 1, statistical significance in patients with POAF was not observed in age (67.7 ± 12.3 vs. 69.2 ± 7.3, *p* = 0.848), gender (67.4 vs. 66.7%, *p* = 0.717), BMI (23.0 ± 3.8 vs. 23.2 ± 4.5, *p* = 0.908), hypertension (45.7 vs. 46.7%, *p* = 0.136), and diabetes (25.0 vs. 40.0%, *p* = 0.426) in this cohort. The chances of taking anti-arrhythmic agents such as beta-blockers were similar in both groups (42.4 vs. 46.7%, *p* = 0.79). POAF developed in 15 patients (15/107 = 14%), of these, 5 out of 57 patients (33.3%) in the dosing ratio of protamine-to-heparin = 1.0 and 10 out of 35 patients (66.7%) in the higher dosing ratios of protamine-to-heparin. Compared to individuals using dosing ratios of protamine-to-heparin >1.0 (up to 1.7), dosing ratios of protamine-to-heparin = 1.0 was associated with reduced risk of POAF (OR = 3.890, CI: 1.130–13.300, *p* = 0.031). As shown in Table 2, when types of diseases (surgeries) were analyzed in terms of higher dosing ratios of protamine-to-heparin, only valvular disorders were significantly associated with POAF (*p* = 0.04).

**Table 1:** Patient demographics and drug effects on POAF.

Characteristics, *n* (%)	No POAF (*n* = 92)	POAF (*n* = 15)	Odds ratio	95% CIP	*P*-value
Age, yr (SD)	67.7 (12.3)	69.2 (7.3)	1.01	0.95–1.06	0.848
Sex, male	67.4% (62)	66.7% (10)	1.26	0.365–4.33	0.717
BMI (SD)	23.0 (3.8)	23.2 (4.5)	0.991	0.851–1.15	0.908
Hypertension	45.7% (42)	46.7% (7)	2.47	0.752–8.14	0.136
Diabetes	25.0% (23)	40.0% (6)	1.67	0.472–5.93	0.426
Medications					
•*β*blocker (*β*, *β* + *α*)	42.4% (39)	46.7% (7)	1.223	0.355–4.12	0.786
•Protamine	92	15			
Dosing ratios of protamine-to-heparin = 1.0	62.0% (57)	33.3% (5)			
Dosing ratios of protamine-to-heparin >1.0 (up to 1,7)	38.8% (35)	66.7% (10)	3.89	1.13–13.30	0.031

POAF, postoperative atrial fibrillation; BMI, body mass index.

**Table 2:** Types of cardiovascular diseases and dosing ratios of protamine-to-heparin.

		Valvular disorders	Ischemic heart disease (CABG only)	Valvular disorders + Ischemic heart disease	Ischemic heart disease + ventricular septal perforation	Aortic dissection	Aortic aneurysm	Others (VSD, ASD)	Patient number
No POAF	Dosing ratios of protamine-to-heparin = 1.0	33	3	4	0	9	4	4	57
	Dosing ratios of protamine-to-heparin >1.0 (up to 1.7)	13	20	0	0	1	0	1	35
POAF	Dosing ratios of protamine-to-heparin = 1.0	4	0	0	0	1	0	0	5
	Dosing ratios of protamine-to-heparin >1.0 (up to 1.7)	0	8	0	1	1	0	0	10
Patient number		50	31	4	1	12	4	5	107

POAF, postoperative atrial fibrillation; CABG, coronary artery bypass graft; VSD, ventricular septal defect; ASD, atrial septal defect.

## Discussion

In this study, we investigated retrospectively the development of POAF after cardiac surgery, based on the recent reports concerning altered Ca^2+^ kinetics in POAF. As for risk factors of POAF, Yamashita et al. recently performed a systematic review and meta-analysis and identified that older age and a history of heart failure were significant risk factors for POAF whether the included studies were prospective or retrospective data sets [12]. Our clinical data suggested that higher dosing ratios of protamine-to-heparin also increased the incidence of POAF.

The advantages of unfractionated heparin are its rapid onset of action, clinical efficacy, rapid neutralization by protamine, safety, and low cost. The dose of heparin used to prevent blood clotting during cardiopulmonary bypass (CPB) is 300–400 U/kg plus additional doses to achieve and maintain an ACT of greater than 480 s [13]. Historically, an ACT target of ≥ 480 s has been considered adequate, although due to the varying measurement methods, there is often a poor correlation between different devices [14]. The individual response to a fixed dose of heparin may vary. Higher doses of heparin may result in better thrombin inhibition, thereby preserving coagulation factors on CPB. Reversing heparin with protamine should be done after separation from CPB. The appropriate dose in relation to the amount of heparin administered is critically important. A common error is to administer additional protamine with ongoing microvascular oozing in the surgical field, despite an absence of evidence that coagulopathy is related to residual heparin [10, 13]. Furthermore, additional protamine can itself have an anticoagulant effect by impairing thrombin generation and potentiating fibrinolysis. The impact of the high protamine-to-heparin ratio on coagulation time was studied by Mittermayr et al. either in-vitro or in the clinical setting [15]. In a joint effort, the European Association for Cardio-Thoracic Surgery (EACTS) and the European Association of Cardiothoracic Anaesthesiology (EACTA) provide evidence-based recommendations in adult-acquired cardiac surgery. It is advised not to exceed a protamine dose in a 1:1 ratio to the initial heparin bolus [16]. Bartoszko et al. also suggested in the recent review article that dosing ratios of protamine-to-heparin have generally been kept no higher than 1:1 [13]. Accumulating evidence supports lower ratios, such as 0.6–1 mg/kg protamine per 100 IU/kg of heparin, as being associated with decreased transfusion and chest drain output [17, 18]. Recently, Lee et al. reported in this journal that they performed a retrospective study of 216 patients who underwent cardiac surgery to search for a safe minimum protamine dose when measuring heparin concentration, using Heparin Management System (HMS) Plus that calculates a patient's heparin dose-responsiveness curve for a target ACT value [19]. When the protamine-to-heparin ratio is set at 1 mg protamine to 100 international unit heparin, they determined that 75% of the calculated total protamine dose is a safe minimum protamine dose to sufficiently neutralize circulating heparin after CPB. On average, this translates into either .37 mg protamine/100 IU heparin of total heparin dose or .54 mg/100 IU of the first heparin bolus [19]. These studies may suggest that a lower protamine-to-heparin ratio is feasible in these clinical settings. Prospective randomized trials may be required to elucidate this issue.

It has been reported that large doses of protamine (>20 mcg/mL) block single skeletal RyR1 channels and inhibit RyR1-mediated Ca^2+^ release from sarcoplasmic reticulum microsomes [6]. Diaz-Sylvester and Copello extended these studies to cardiac RyR2 reconstituted into planar lipid bilayers [8]. They found that protamine (0.02–20 mcg/mL) added to the cytosolic surface of fully activated RyR2 affected channel activity in a voltage-dependent manner. They observed a full block of cardiac RyR2 in the presence of 20 mcg/mL protamine. Protamine did not simply block RyR2 in an all-or-none fashion. Rather, it affected the RyR2 channel conduction pathway by producing substates and full blocks in a voltage-dependent and dose-dependent manner. The addition of heparin (250 mcg/mL), known to bind protamine with very high affinity, reversed the effect of protamine on RyR2. This reversibility suggests that the action of protamine was not related to the dissociation of any cofactor or modulatory subunit bound to the RyR2 channel. Their results open the possibility that direct protamine binding to RyR2 could mediate, at least in part, the observed abnormalities in SR Ca^2+^ homeostasis [8]. Our clinical observation may support theirs in vitro results.

Adverse effects of protamine include anaphylactic reactions caused by an injection of protamine during cardiac surgery are well-known complications [17, 20]. Gertler et al. observed that platelet function worsens with protamine to heparin ratio higher than 1:1 in blood samples taken from ten healthy volunteers [21]. Similar impairment of platelet aggregation and function with the use of protamine to heparin ratio above 2.6:1, was demonstrated by Carr and Silverman [22]. Protamine has also been shown to display immunological and inflammatory alterations [17].

**Figure 1:**
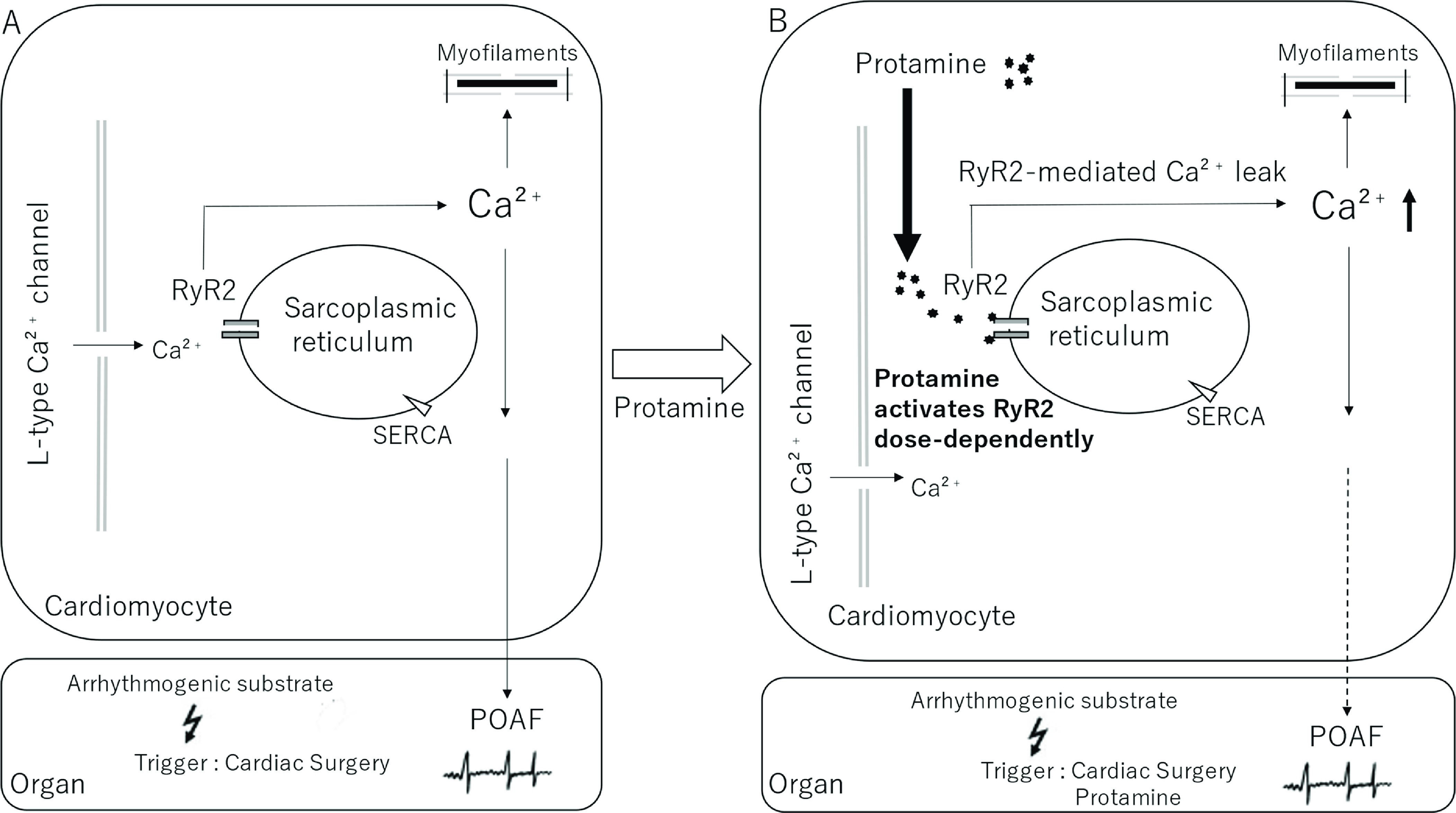
A hypothetical model illustrating the Ca^2+^ signaling pathways implicated in POAF. Cytosolic Ca^2+^ handling abnormalities in atrial myocytes have been suggested to play an important role in the initiation and maintenance of atrial fibrillation. (A) Fakuade et al. reported that sarcoplasmic reticulum (SR) Ca^2+^ leak and ryanodine receptor channel (RyR2) function in atrial myocytes. Reduced SERCA-mediated Ca^2+^ reuptake into the SR is a major contributor to impaired preoperative atrial contractile function and the pre-existing arrhythmogenic substrate in patients developing POAF. Therefore, modulation of SERCA activity may represent a novel mechanistic target to prevent the development of POAF. (B) With higher dosing ratios of protamine-to-heparin, the residual protamine may activate RyR2 dose-dependently, followed by RyR2-mediated Ca^2+^ leak, resulting in increased cytosolic Ca^2+^ and POAF. Therefore, dosing ratios of protamine-to-heparin may be an important determinant in the development of POAF in addition to reduced SERCA-mediated Ca^2+^ reuptake into the SR described in Figure 1A. POAF, postoperative atrial fibrillation; SR, sarcoplasmic reticulum; SERCA, sarcoplasmic reticulum calcium ATPase (sarcoplasmic reticulum calcium pump).

POAF, defined as new-onset AF in the immediate postoperative period, is a clinically relevant problem, occurring in 20–40% of patients after cardiac surgery, with the peak incidence between postoperative days 2 and 4 [1]. Intra- and postoperative changes affecting AF triggers and pre-existing atrial substrate may increase atrial vulnerability to AF. Many episodes of POAF are self-terminating and some are asymptomatic, but POAF has been associated with a four- to the five-fold risk of recurrent AF in the next 5 years. It has also been shown to be a risk factor for stroke, myocardial infarction, and death compared with non-postoperative AF patients [3, 4]. The pathogenesis of POAF may be multifactorial. β-Adrenergic stimulation has been implicated in the pathogenesis of POAF [23]. Some surgeries are more likely to induce POAF [24]. Specifically, patients who undergo combined surgeries, such as combined valve and coronary artery bypass graft (CABG) surgeries, are twice as likely to develop POAF, up to 60% [25]. When types of surgery (diseases) were analyzed in terms of higher dosing ratios of protamine-to-heparin (Table 2), only valvular disorders were marginally associated with POAF (*p* = 0.04). In Figure 1A, Fakuade et al. provide the evidence in vitro that impaired SR Ca^2+^ uptake is a common underlying mechanism that contributes both to the impaired pre-existing atrial contractile function as an independent risk factor of POAF and to the arrhythmogenic substrate, which predisposes patients to the development of POAF [5]. Based on the clinical results, we propose a hypothetical model of protamine-related POAF in Figure 1B. In the case of higher dosing ratios of protamine-to-heparin, the residual protamine may activate RyR2 dose-dependently, followed by RyR2-mediated Ca^2+^ leak, resulting in increased cytosolic Ca^2+^ and POAF. In the current retrospective study, POAF developed both in 5 of 57 patients (33.3%) in the dosing ratio of protamine-to-heparin = 1.0 and 10 out of 35 patients (66.7%) in the higher dosing ratios of protamine-to-heparin (Table 2). Statistical analysis showed that POAF was associated with higher dosing ratios of protamine-to-heparin >1.0 up to 1.7 and was statistically significant (odds ratio = 3.890, 95% CI = 1.130–13.300, *p*-value = 0.031). POAF development in the dosing ratio of protamine-to-heparin = 1.0 may suggest that residual protamine has some adverse effect on inducing POAF. If this is the case, a lower protamine-to-heparin ratio (less than protamine-to-heparin = 1.0) may be used to prevent POAF, as discussed above [19]. Further studies are needed to elucidate this issue.

## Limitations

The study has some limitations. First, this small study is a retrospective analysis of an integrated database from a secondary medical center in the Iga district, Mie, Japan. Second, AF was identified by diagnostic code, electrocardiogram, and medical history, not by a long-term recorder, some patients with AF might be underdiagnosed. Furthermore, although potential arrhythmogenic mechanisms for POAF development may be associated with higher dosing ratios of protamine-to-heparin as shown in this study, there may be additional factors predisposing to the development of POAF, including genetic, type of surgery, autonomic nervous system, inflammation, and pre-existing fibrosis.

## Conclusion

In conclusion, our results provide evidence that higher dosing ratios of protamine-to-heparin may increase the incidence of POAF. In this regard, STS/SCA/AmSECT and EACTS/EACTA clinical guidelines on anticoagulation during cardiopulmonary bypass are useful in clinical practice.

## Data Availability

All available data are incorporated into the article.
